# Exploring anti-acute kidney injury mechanism of Dahuang-Gancao decoction by network pharmacology and experimental validation

**DOI:** 10.18632/aging.205033

**Published:** 2023-09-18

**Authors:** Rui Wang, Yi An, Yifang Xu, Chengyin Li, Qiyuan Wang, Yinshui Zou, Guangzhi Wang

**Affiliations:** 1 Department of Oncology, Hubei Provincial Hospital of Traditional Chinese Medicine, Wuhan 430065, P.R. China; 2 Department of Endocrinology, Second Affiliated Hospital of Wuhan University of Science and Technology, Wuhan 430065, P.R. China; 3 Department of General Surgery, The Second Hospital of Dalian Medical University, Dalian 116023, P.R. China

**Keywords:** acute kidney injury, network pharmacology, Dahuang-Gancao decoction, experimental validation, SIRT3/NRF2/HO-1 signaling pathway

## Abstract

This study aimed to investigate the pharmacological effects and molecular mechanisms of Dahuang-Gancao Decoction (DHGC) on acute kidney injury (AKI). Network pharmacology was utilized to analyze the key targets of DHGC against AKI. These targets were used to construct a protein-protein interaction (PPI) network, which was analyzed using Gene Ontology (GO) and Kyoto Encyclopedia of Genes and Genomes (KEGG) enrichment to predict the mechanism of action. Based on the network pharmacological analysis, Sirtuin 3 (SIRT3) was identified as a key target, and apoptosis was suggested as a mechanism of DHGC for AKI treatment. Subsequently, an AKI mouse model was induced using lipopolysaccharide (LPS), and the study demonstrated that DHGC gradient intervention significantly reduced plasma urea and creatinine levels in AKI mice, ameliorated renal pathological changes, reduced apoptosis, and lowered serum inflammatory factors. The mechanism of DHGC’s anti-AKI effect may lie in the activation of the SIRT3/NRF2/HO-1 signaling pathway, which plays an antiapoptotic role in renal cells. In summary, DHGC improved LPS-induced AKI in mice by activating the SIRT3/NRF2/HO-1 signaling pathway. These findings shed light on the potential clinical application of DHGC for the treatment of nephropathy.

## INTRODUCTION

Acute kidney injury (AKI) is a clinical condition characterized by a rapid decline in renal excretory function, accumulation of nitrogen metabolites, reduced urine output, metabolic acidosis, and elevated potassium and phosphorus concentrations [1, 2]. Recent statistics indicate a high incidence of AKI globally, with more than 5,000 cases per million people per year of non-dialysis AKI and 295 cases per million people per year requiring dialysis, and a high overall mortality rate [3]. The major pathological changes associated with AKI include fluid, electrolyte, and acid-base imbalances, as well as damage to multiple organs throughout the body. As AKI progresses, the majority of kidney units may become irreversibly destroyed and lost, leading to end-stage renal disease [4]. Unfortunately, there are currently no effective treatments for AKI that can reduce kidney damage or accelerate kidney unit recovery. Therefore, exploring effective pharmacological therapies is of great clinical importance.

Traditional Chinese Medicine (TCM) has been widely used for thousands of years in China to treat kidney disease and its complications [5]. Among the TCM formulas, Dahuang-Gancao Decoction (DHGC), which contains *Rheum palmatum L* (rhubarb) and *Radix glycyrrhiza* (licorice), is a well-known Chinese medicine formula used to treat kidney disease. DHGC was first recorded in a famous ancient medical book "Treatise on Cold Pathogenic and Miscellaneous Diseases" in the Han Dynasty, and it can be used for the treatment of constipation, excess heat stagnation of the gastrointestinal tract, and related symptoms [6]. According to TCM theory, licorice can alleviate rhubarb’s drastic nature, making DHGC more suitable for treating AKI. Modern pharmacological studies have shown that Rhubarb and licorice play an important role in the treatment of AKI [7, 8]. For example, Rhubarb and its active components possess anti-inflammatory, antioxidant, and antiapoptotic effects, which can effectively delay the progression of AKI [2, 9]. Licorice can alleviate methotrexate-induced AKI by decreasing oxidative stress and suppressing the ensuing activation of proapoptotic and proinflammatory pathways [10]. This predicts that DHGC has a therapeutic effect on AKI. However, the potential pharmacological mechanisms underlying the therapeutic effects of DHGC and its components in the treatment of AKI are still unclear, and additional preclinical evidence is needed.

With advances in bioinformatics, systems biology, and pharmacology, network pharmacology has emerged as a new strategy to elucidate the active ingredients and potential mechanisms of herbal compounds [5]. Network pharmacology analyzes the network of biological systems and selects specific signaling nodes to design multitarget drug molecules, which is in line with the characteristics of Chinese medicine, which often employs multi-component, multitarget, and compound actions [11]. Therefore, this study aims to identify the potential molecular mechanisms of DHGC against AKI using network pharmacology to provide a basis for further animal experiments. Furthermore, a mouse experiment is designed to validate the crucial role of the molecular mechanism of DHGC against AKI. This study is expected to reveal the potential mechanism of DHGC in treating AKI and provide a new strategy for studying drugs to treat AKI.

## MATERIALS AND METHODS

### Collection and screening of the main chemical components and targets of DHGC

The traditional Chinese medicine systems pharmacology database and analysis platform (TCMSP) was utilized to identify compounds and targets related to DHGC. Candidate compounds were selected based on drug-likeness (DL) values ≥0.18 and oral bioavailability (OB) ≥30%. Targets of the candidate rhubarb and licorice compounds were obtained from the TCMSP database, and translated into corresponding gene names using the protein database UniProt.

### AKI disease target collection and Venn diagram construction

The GeneCards, OMIM, and DrugBank databases were searched using the keywords “Acute kidney injury” to identify human genes associated with AKI. Repetitive targets were removed, and all target genes were converted to human genes using the UniProt database. The DHGC-related targets and AKI-related targets were analyzed using Omicshare to generate a Venn diagram to determine the intersected targets.

### Constructing a “DHGC-Active ingredient-target” network and protein-protein interaction (PPI) network

The “DHGC-Active Ingredient-Target” network was constructed using Cytoscape 3.8.2 to elucidate the pharmacological mechanisms of action of DHGC. The intersected targets were imported into the STRING 11.0 database, and a PPI network model was constructed using “Homo sapiens” as the biological species set, with a confidence score >0.4 and unconnected nodes hidden. The resulting proteins were introduced into Cytoscape 3.8.2 software to construct the PPI networks, and node connectivity was analyzed to identify core targets.

### Gene ontology (GO) functional annotation and Kyoto Encyclopedia of Genes and Genomes (KEGG) pathway analysis

GO functional enrichment analysis and KEGG pathway enrichment analysis were conducted for the common targets of DHGC and AKI using R software and the DAVID database, with a threshold value of *P* < 0.05. DHGC was found to interfere with the biological pathway of AKI. Bubble plots were generated to display the top 20 items of GO analysis and the top 20 items of KEGG analysis.

### Reagents

Rhubarb and licorice were purchased from Hubei Tianji Chinese Medicine Decoction Company (Wuhan, China). The primary antibody against SIRT3 was obtained from Cell Signaling Technology Inc., (#2627, Beverly, MA, USA), while other antibodies, including NRF-2 (16396-1-AP), HO-1 (10701-1-AP), and β-actin (81115-1-RR), were procured from Proteintech Group, Inc. (Wuhan, China). DHGC was prepared in accordance with the guidelines set forth in the “Treatise on Cold Pathogenic and Miscellaneous Diseases.” In brief, the drug-solvent ratio was 1:10, with 40 g of Rhubarb species (*Rheum palmatum L*) and 10 g of licorice species (*Radix glycyrrhizae*) added to 500 ml of boiling water. The herbs were subjected to reflux extraction with water for 1.5 h twice to obtain the water decoction and concentrate. The DHGC decoction was then dried in a vacuum drier to calculate the extraction rate, and the ratio of DHGC decoction powder to raw herbs was 15.61%. The resulting dry extract was stored at −20°C until use.

### Animal experiment

Male C57BL/6 mice (six weeks old, 20 ± 2 g) were obtained from Hubei Center for Disease Control and Prevention (Wuhan, China). Mice were adaptively housed for one week under a 12 h light/dark cycle (23 ± 2°C, 55% ± 5% humidity) with free access to food and water. The mice were randomly divided into five groups (*n* = 8): Ctrl group, AKI group, AKI+ low dose group (Low group), AKI+ medium dose group (Medium group), and AKI+ high dose group (High group). DHGC water extract (50 mg/kg, 100 mg/kg, 150 mg/kg) was administered to the Low, Medium, and High groups for three days before LPS administration. Then, the AKI group, Low group, Medium group, and High group were injected with LPS (10 mg/kg, i.p.), while the Ctrl group received an equal volume of saline (i.p.). After 24 hours, all mice were euthanized, and the kidney and serum were collected. Kidney tissues were photographed, and all samples were stored at −80°C for further experiments. The animal experiments were performed in accordance with the guidelines set forth by the Animal Care and Use Committee of the animal facility at the Hubei University of Chinese Medicine (No: HUCMS202302007).

### Analysis of creatinine and urea

The Creatinine Colorimetric Assay Kit and Urea Colorimetric Assay Kit were utilized to determine the levels of creatinine and urea, respectively, in serum, as per the manufacturer’s instructions (E-BC-K188-M and E-BC-K183-M, Elabscience Biotechnology Co., Ltd, Wuhan, China).

### Histological analysis

Kidney tissues were fixed with 4% paraformaldehyde, dehydrated, paraffin-embedded, and sectioned into 5 μm-thick sections. Deparaffinization was performed using xylene and diluted ethanol. Hematoxylin and eosin (H&E) staining was applied, and the TUNEL Staining Kit (E-CK-A320, Elabscience Biotechnology Co., Ltd, Wuhan, China) was employed to assess apoptosis levels. Images were captured using a Leica DFC310 FX digital camera coupled with a Leica DMI4000B light microscope (Wetzlar, Germany).

### RNA extraction and real-time quantitative PCR (RT-qPCR)

Based on the manufacturer’s protocol, total RNA of kidney was extracted using Trizol reagent and reversely transcribed to cDNA with a first-strand cDNA synthesis kit (G3337, Servicebio, Wuhan, China). The relative mRNA levels of target genes were measured by RT-qPCR using a SYBR QPCR mixture (AM2103, Allmeek Co., Ltd, Beijing, China) at the ABI 7500 Real-Time Fluorescence Quantitative PCR instrument. The thermal cycle condition was as follows: pre-denaturing at 95°C for 10 min; 40 cycles of denaturation at 95°C for 10 s, annealing/extension at 60°C for 30 s. Target gene expressions were normalized against that of β-actin, and fold changes were calculated using a 2(^−ΔΔCT^) method. The list of PCR primers used in this study is shown in Table 1.

**Table 1:** Primer sequence list for RT-PCR analysis.

**Gene**	**Forward primer (5′–3′)**	**Reverse primer (5′–3′)**
SIRT3	ATCCCGGACTTCAGATCCCC	CAACATGAAAAAGGGCTTGGG
NRF2	CACATCCAGTCAGAAACCAGTGG	GGAATGTCTGCGCCAAAAGCTG
HO-1	GAACCCAGTCTATGCCCCAC	GGCGTGCAAGGGATGATTTC
β-Actin	GGCTGTATTCCCCTCCATCG	CCAGTTGGTAACAATGCCATGT

### Western blot

Total protein was extracted from kidney tissues using RIPA buffer (G2033, Servicebio, Wuhan, China), along with a protease inhibitor cocktail (G2006, Servicebio, Wuhan, China). The protein concentration was determined using a bicinchoninic acid (BCA) protein assay kit (G2026, Servicebio, Wuhan, China). The protein samples were separated on sodium dodecyl sulfate-polyacrylamide gel electrophoresis (SDS-PAGE) gels and transferred onto polyvinylidene difluoride (PVDF) membranes. After blocking with 5% skim milk in Tris-buffered saline tween-20 (TBST) for 1 hour, the membranes were incubated separately with primary antibodies at 4°C overnight, including SIRT3, NRF2, HO-1, and β-actin. After washing with TBST, the membranes were incubated with a secondary antibody conjugated with horseradish peroxidase (HRP) for 1.5 hours. The protein signals were finally visualized using an ECL Protein Detection kit.

### Statistical analysis

The data are expressed as the mean ± SD. An unpaired two-tailed Student’s *t*-test was used to compare the difference between the two groups, while one-way ANOVA was used to assess differences among multiple groups. *P* < 0.05 was considered statistically significant. GraphPad Prism (Version 8.0.1, GraphPad Software Inc., CA, USA) was utilized for regular analysis.

### Data availability statement

The data used to support the findings of this study are available from the corresponding author upon request.

## RESULTS

### Composition and target screening of DHGC

In the screening process, a total of 108 chemical components were identified in DHGC. The top 20 components are listed in Table 2. To identify protein targets associated with DHGC, we utilized the TCMSP database and subjected the resulting targets to normalization using the Uniprot database. This process yielded a total of 3,161 gene targets, from which 2,991 duplicate targets were eliminated, leaving a final count of 170 unique targets.

**Table 2:** Active components of GGQL (top 20 of OB).

**ID**	**Active components**	**OB (%)**	**DL**
MOL002311	Glycyrol	90.78	0.67
MOL004990	7,2′,4′-trihydroxy—5-methoxy-3—arylcoumarin	83.71	0.27
MOL000471	Aloe-emodin	83.38	0.24
MOL004904	Licopyranocoumarin	80.36	0.65
MOL004891	Shinpterocarpin	80.3	0.73
MOL005017	Phaseol	78.77	0.58
MOL004841	Licochalcone B	76.76	0.19
MOL004810	Glyasperin F	75.84	0.54
MOL001484	Inermine	75.18	0.54
MOL000500	Vestitol	74.66	0.21
MOL005007	Glyasperins M	72.67	0.59
MOL004941	(2R)-7-hydroxy-2-(4-hydroxyphenyl) chroman-4-one	71.12	0.18
MOL004959	1-Methoxyphaseollidin	69.98	0.64
MOL000392	Formononetin	69.67	0.21
MOL004863	3-(3,4-dihydroxyphenyl)-5,7-dihydroxy-8-(3-methylbut-2-enyl) chromone	66.37	0.41
MOL004903	Liquiritin	65.69	0.74
MOL004808	Glyasperin B	65.22	0.44
MOL004829	Glepidotin B	64.46	0.34
MOL004855	Licoricone	63.58	0.47
MOL004914	1,3-dihydroxy-8,9-dimethoxy-6-benzofurano (3,2-c) chromenone	62.9	0.53

### AKI intersectional targets and the “DHGC-AKI” database

**Figure 1:**
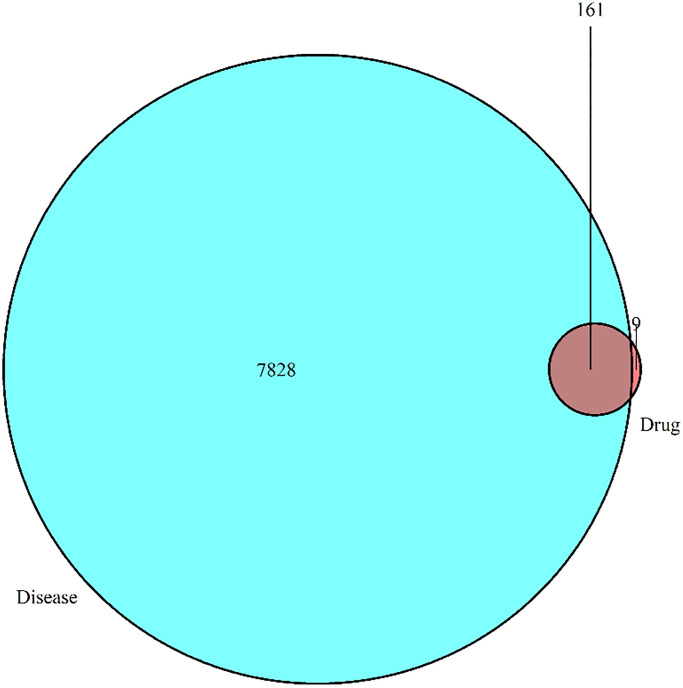
**Venn diagram for drug target and disease target screening.** After preliminary screening, 7,828 targets were identified for AKI, 170 for DHGC, and 161 for DHGC and AKI.

In order to elucidate the mechanism and pharmacodynamics of DHGC, we extracted a total of 7,828 target genes associated with AKI from GeneCard, OMIM, and DrugBank databases. By performing a Venn diagram analysis, we identified 161 unique targets that are common to both DHGC and AKI, as depicted in Figure 1.

### Ingredient-target-disease network

**Figure 2:**
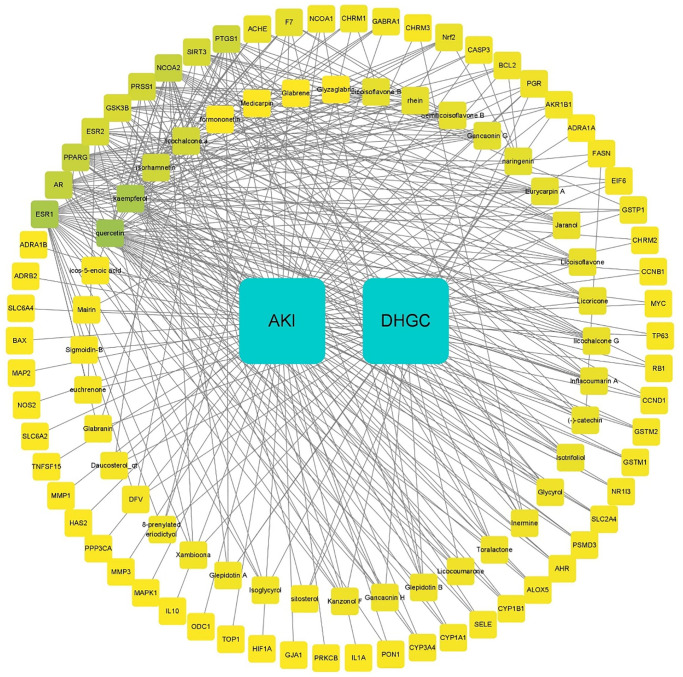
**Interaction network of DHGC compound-AKI targets.** The blue squares represent DHGC and AKI, the inner circle represents the active components, and the outer circle represents the target genes of the DHGC compound acting on AKI, which are divided by degree centrality (DC) values. Blue indicates a higher DC value. The number of edges connected to the node in the network represents the degree of freedom.

To investigate the association between active ingredients, potential targets, and AKI, we constructed an ingredient-target-disease network using Cytoscape 3.8.2 software. The network consisted of 10 nodes and 32 edges, as shown in Figure 2. The top 10 key targets of DHGC in the treatment of AKI were identified as ESR1, AR, PPARG, ESR2, GSK3β, PRSS1, NCOA2, SIRT3, PTGS1, and ACHE.

### PPI network construction

**Figure 3:**
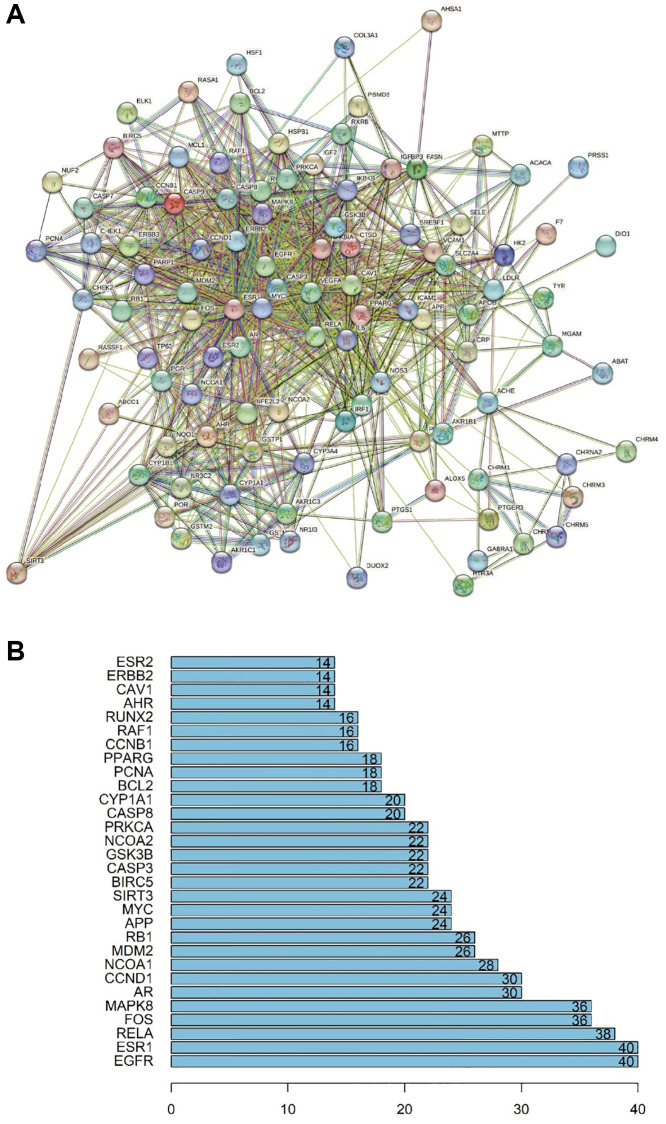
**PPI network.** (**A**) Network nodes represent proteins: colored nodes represent query proteins and the first shell of interactors; white nodes represent the second shell of interactors; empty nodes represent proteins of unknown 3D structure; filled nodes represent some 3D structure that is known or predicted. Edges represent protein–protein associations: the light blue edges represent curated databases; the fuchsia edges represent experimentally determined associations; the green edges represent gene neighborhoods; the red edges represent gene fusions; the dark blue edges represent gene co-occurrence; the light green edges represent text mining; the black edges represent co-expression; the light purple edges represent protein homology. The thickness of the line in the figure represents the strength of the force. (**B**) Statistics of the most multilateral nodes of the PPI network.

To identify the core proteins targeted by DHGC for the intervention of AKI, we constructed a protein-protein interaction (PPI) network consisting of 110 nodes and 1008 edges using the STRING database (Figure 3A). After removing targets that were not linked to other targets, we further analyzed the nodes with the highest number of edges, as illustrated in Figure 3B.

### GO enrichment and KEGG pathway analyses

**Figure 4:**
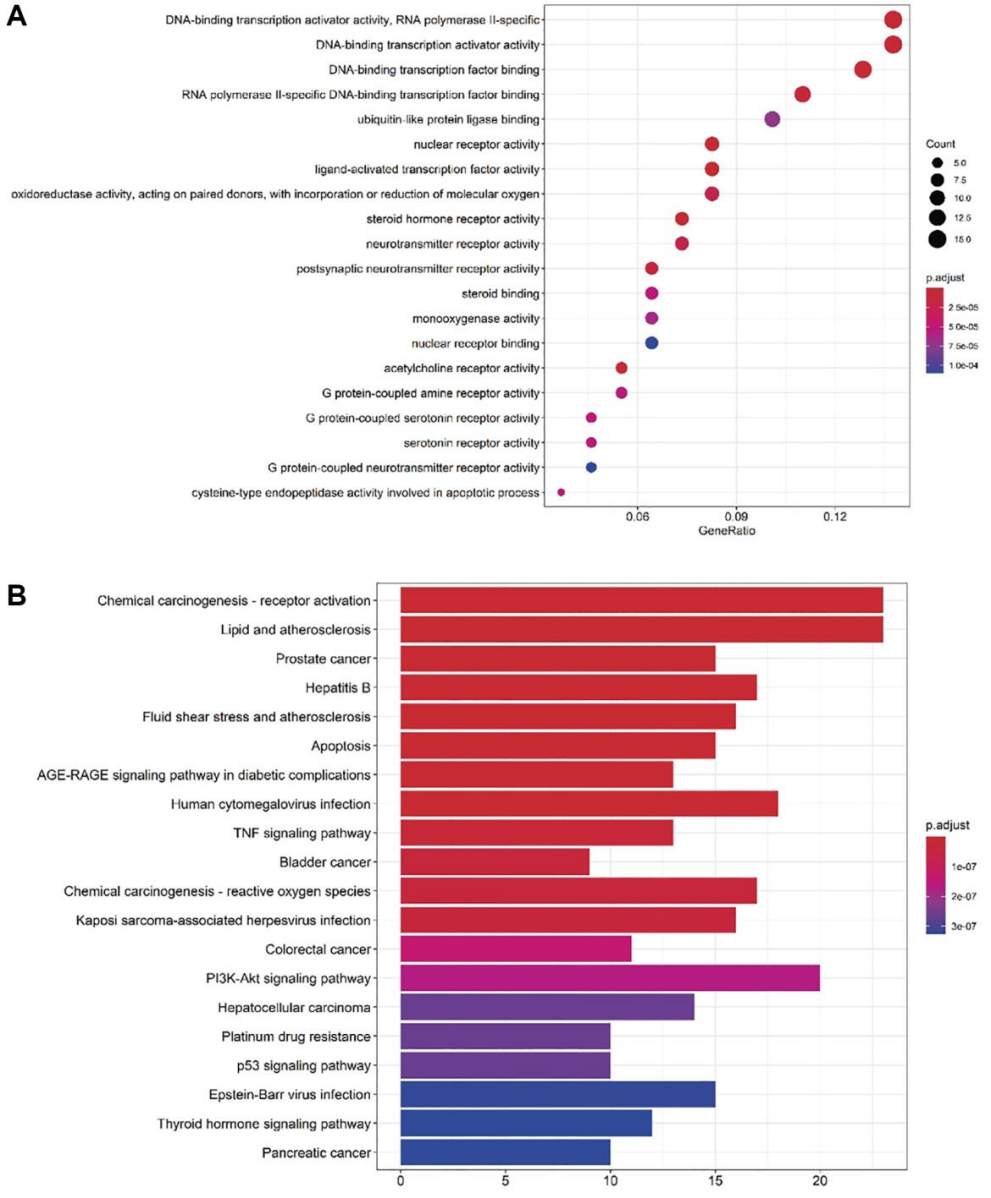
**GO enrichment analysis and KEGG enrichment analysis of DHGC anti-AKI.** (**A**) GO analysis of core targets (top 20). (**B**) Pathways of the DHGC compound against AKI (top 20).

To elucidate the molecular mechanisms underlying DHGC’s therapeutic effects against AKI, we performed GO enrichment and KEGG pathway analyses. The clusterProfiler package of R language software was used to conduct GO enrichment analysis on the 161 therapeutic targets of DHGC against AKI, resulting in a total of 114 significant GO entries (*P*-value < 0.05). The top entries included RNA polymerase II-specific DNA-binding transcription activator activity, DNA-binding transcription factor binding, DNA-binding transcription activator activity, RNA polymerase II-specific DNA-binding transcription factor binding, ubiquitin-like protein ligase binding, and nuclear receptor activity, among others (Figure 4A).

We also performed KEGG pathway analysis using the clusterProfiler package, with human as the selected species (organization = “has”) and *P*-value < 0.5 as the threshold. A total of 122 pathways were obtained, with the top 20 ranked based on the correlation and *P*-value. The pathways included chemical carcinogenesis-receptor activation, lipid and atherosclerosis, prostate cancer, hepatitis B, fluid shear stress and atherosclerosis, and apoptosis. Notably, the signaling pathways involving inflammation, oxidative stress, and apoptosis were highly correlated (Figure 4B).

### DHGC ameliorated physiochemical parameters in AKI mice

**Figure 5:**
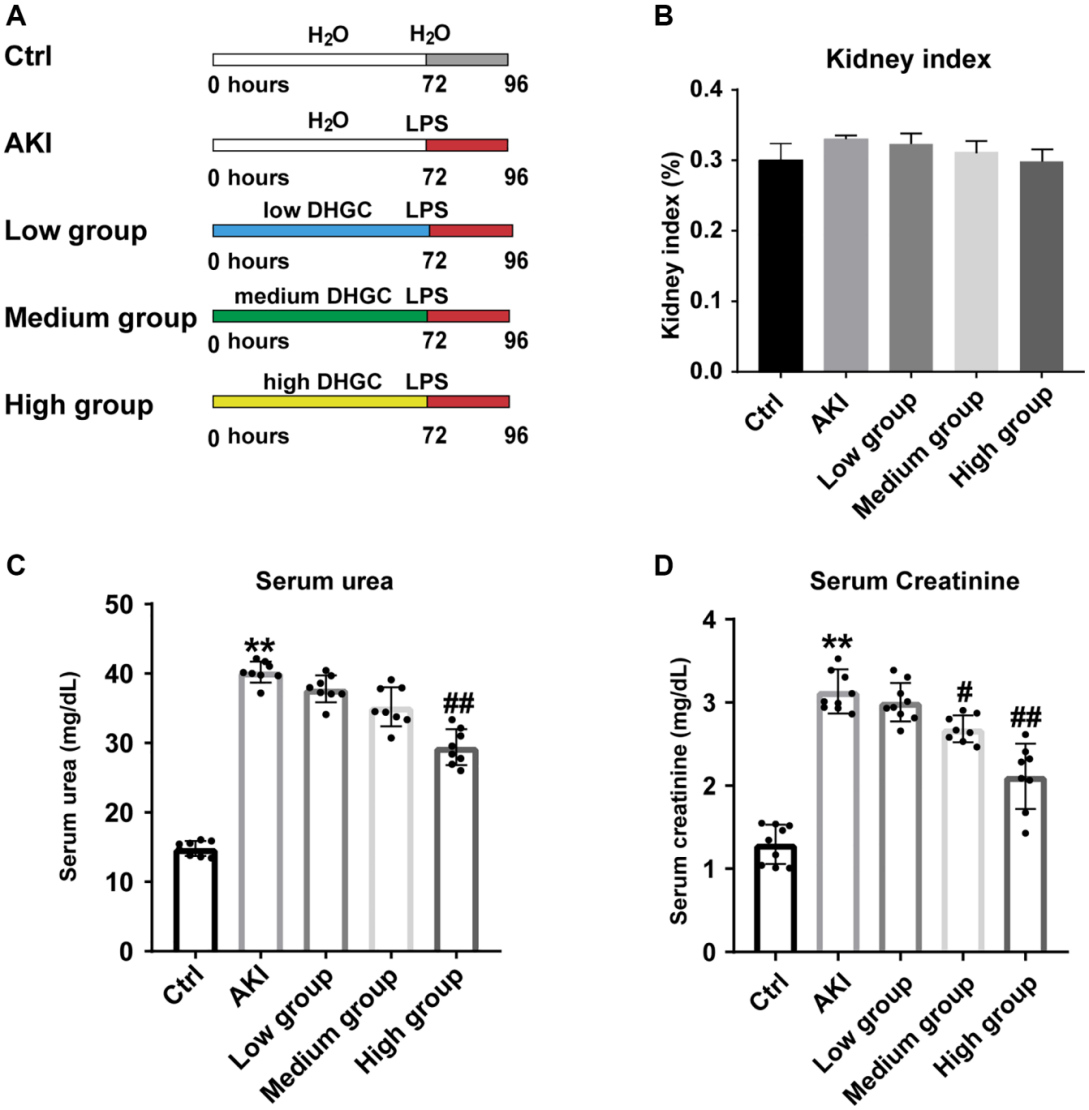
**Improvement of physiochemical parameters in LPS-induced AKI mice by DHGC.** (**A**) Experimental schematic diagram. (**B**) Kidney index. (**C**) Level of serum urea. (**D**) Level of serum creatinine. Data are presented as the mean ± SD (*n* = 6). ^*^P < 0.05, ^**^P < 0.01 vs. Ctrl group; ^#^*P* < 0.05, ^##^*P* < 0.01 vs. AKI group.

A schematic diagram of the animal experiment is depicted in Figure 5A. Briefly, mice were treated with DHGC for three days and then induced with AKI by intraperitoneal injection of LPS (10 mg/kg). There was no significant difference in kidney index among all groups (Figure 5B). Additionally, DHGC administration at a high dosage suppressed the elevation of urea and creatinine levels in AKI mice (*P* < 0.01, vs. AKI group) (Figure 5C, 5D).

### DHGC mitigates renal injury and apoptosis in AKI mice

**Figure 6:**
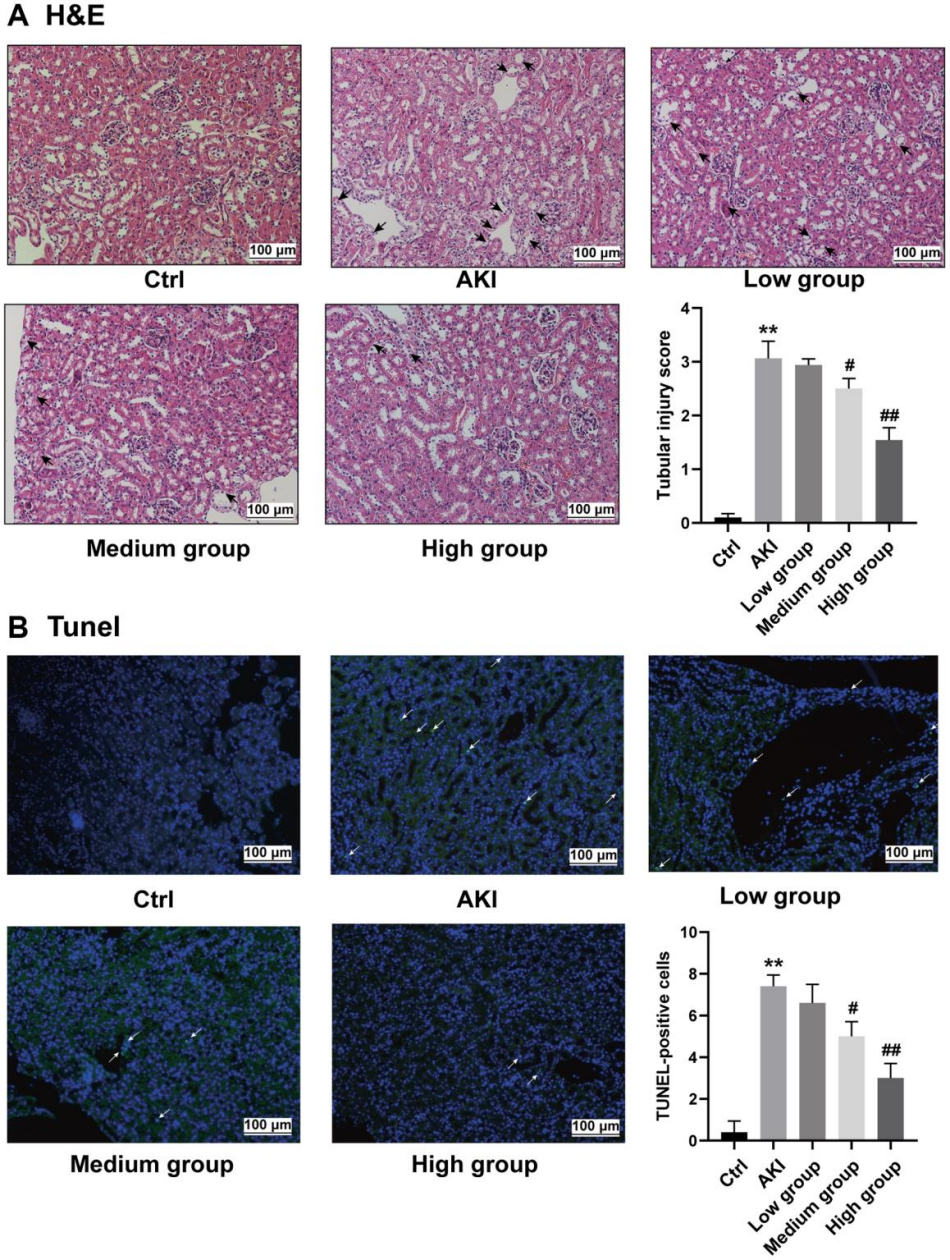
**Reversal of renal pathological injury and reduced apoptosis in AKI mice by DHGC.** (**A**) Representative histology and pathological tubular injury score in the renal cortex by H-E staining (200×, arrows represent renal tubular epithelial injury). (**B**) Apoptosis was also evaluated by TUNEL staining and quantification of TUNEL-positive cells (200×, arrows indicate the apoptosis-positive area).

The renoprotective effect of DHGC against renal injury was demonstrated through H&E staining of the kidneys. Mice subjected to LPS injection exhibited noticeable edema in the renal tubular epithelial cells, which was prevented in all DHGC-treated groups. The renal tubule injury score was gradually reduced after DHGC gradient intervention (*P* < 0.01, vs. AKI group) (Figure 6A). Additionally, LPS-induced injury resulted in a significant increase in the number of TUNEL-positive cells in kidney tissue, while DHGC treatment led to a significant reduction in apoptotic cells in kidney tissue samples. TUNEL-positive cells were significantly reduced after DHGC intervention (*P* < 0.01, vs. AKI group) (Figure 6B).

### DHGC decreases serum inflammatory factors in AKI mice

**Figure 7:**
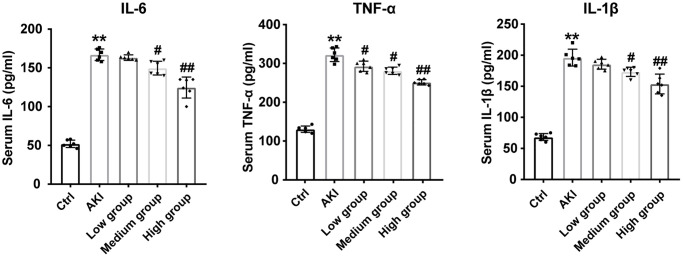
**DHGC reduces inflammation in AKI mice.** Serum IL-6, TNF-α, and IL-1β levels. Data are presented as the mean ± SD (*n* = 6). ^*^P < 0.05, ^**^P < 0.01 vs. Ctrl group; ^#^*P* < 0.05, ^##^*P* < 0.01 vs. AKI group.

Elevated levels of proinflammatory cytokines are a hallmark of acute kidney injury [12]. To investigate whether DHGC affects the inflammatory response, we measured the levels of these cytokines in serum. As shown in Figure 7, IL-6, TNF-α, and IL-1β levels were significantly increased in the AKI group, whereas DHGC intervention significantly reduced these cytokine levels. These findings suggest that DHGC effectively attenuated the LPS-induced inflammatory response.

### DHGC effects on the SIRT3/NRF2/HO-1 signaling pathway in AKI mice

**Figure 8:**
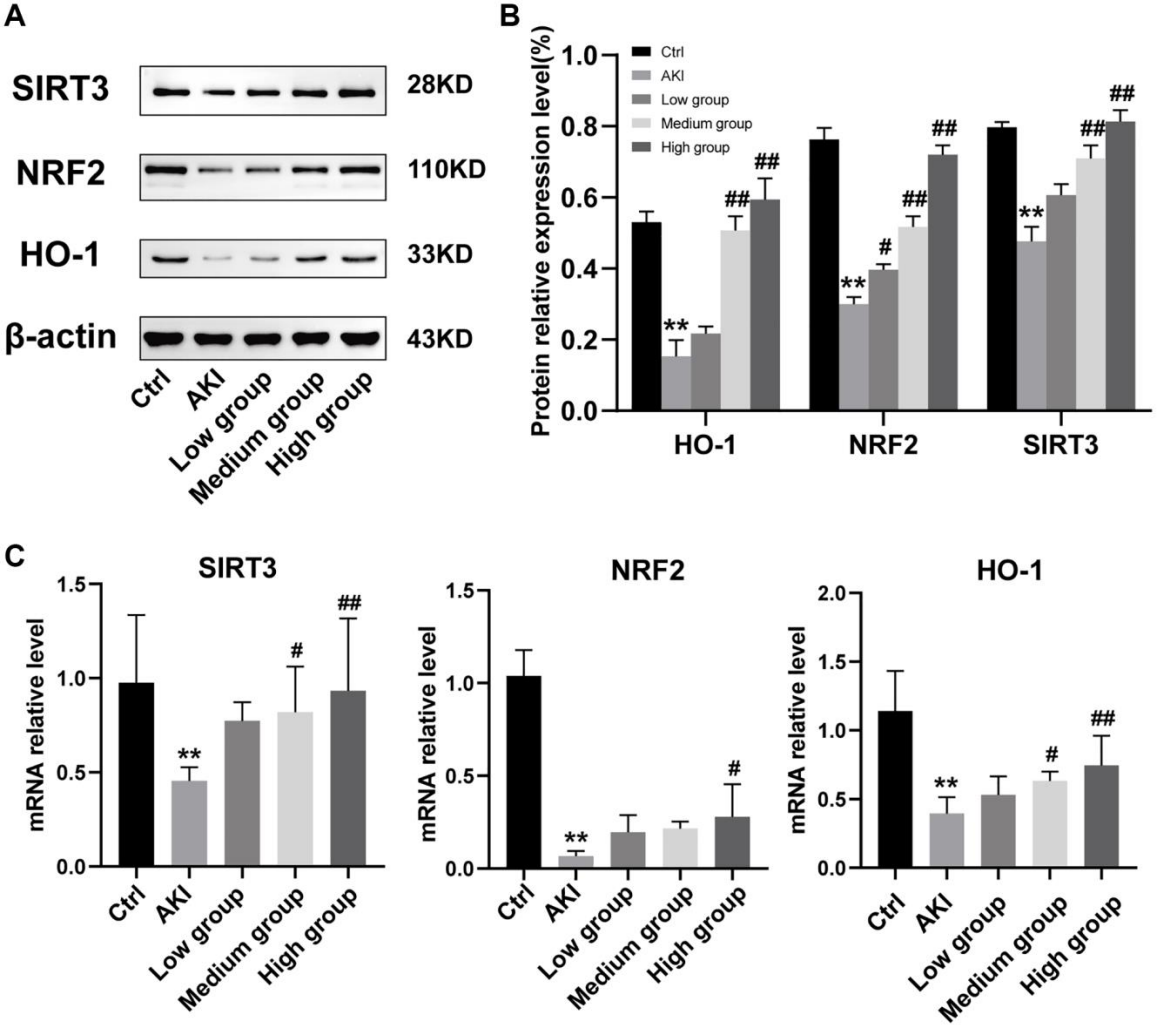
**Expression of the SIRT3/NRF2/HO-1 signaling pathway.** (**A**) Representative images of the effect of DHGC on the SIRT3/NRF2/HO-1 signaling pathway in AKI mice by Western blot. (**B**) Statistical analysis of the results of the Western blot. (**C**) Expression of SIRT3, NRF2, and HO-1 in kidney tissues at mRNA level. Data are presented as the mean ± SD. ^**^P < 0.01 vs. Ctrl group; ^#^*P* < 0.05, ^##^*P* < 0.01 vs. AKI group.

To gain further insight into the effect of DHGC on the SIRT3/NRF2/HO-1 signaling pathway, we conducted western blot analysis to examine the protein expression of SIRT3, NRF2, and HO-1 in the kidneys. Our findings demonstrated that SIRT3, NRF2, and HO-1 expression were significantly reduced in AKI mice (Figure 8A, 8B). In contrast, treatment with DHGC remarkably suppressed the protein alterations in these regulators and kinases in AKI mice, suggesting a protective effect of DHGC on the SIRT3/NRF2/HO-1 signaling pathway. SIRT3, NRF2, and HO-1 at the mRNA level, DHGC also showed the same effect (*P* < 0.01 and 0.05, vs. AKI group) (Figure 8C).

## DISCUSSION

Acute kidney injury (AKI) is a critical clinical condition primarily managed through symptomatic therapy, as specific treatment strategies have yet to be identified. Nonetheless, herbal formulations have a long-standing history of aiding in the treatment of AKI [13]. In this study, we utilized network pharmacology to investigate the potential molecular mechanism underlying the anti-AKI effects of DHGC, a Chinese medicine formula. Our network pharmacological findings were further corroborated through animal experiments.

Through basic network pharmacological analysis, we identified the main targets of DHGC against AKI to be EGFR, ESR1, MAPK8, AR, CCND1, NCOA1, MDM2, RB1, APP, MYC, and SIRT3. Some of these targets facilitate AKI, such as EGFR, which accelerates renal cell apoptosis and caspase-3 activation during AKI progression [14, 15]. On the other hand, several targets play a protective role against AKI, including SIRT3, which inhibits kidney cell apoptosis caused by mitochondrial damage, thereby delaying the progression of AKI [16, 17]. Our analysis suggests that one of the mechanisms by which DHGC exerts its anti-AKI effects is by inhibiting renal cell apoptosis through the attenuation of mitochondrial damage. Mitochondrial damage is a significant mechanism underlying apoptosis induction in kidney cells, and our research shows that DHGC’s active ingredients have protective effects against mitochondrial damage. For instance, quercetin, the major chemical constituent in DHGC, modulates pathways associated with mitochondrial biogenesis, ATP anabolism, and intramitochondrial redox status, ultimately inhibiting mitochondria-induced apoptosis [18]. Additionally, aloe-emodin can reduce mitochondrial damage and inhibit the caspase-3 apoptotic signaling pathway [18]. Our KEGG enrichment analysis also supports the hypothesis that the anti-AKI action of DHGC is linked to the inhibition of renal apoptosis through the attenuation of mitochondrial damage. Specifically, we found that apoptosis was highly correlated, and further analysis showed that SIRT3, a critical target of DHGC against AKI that plays a role in maintaining mitochondrial homeostasis, is a key factor in this process [19]. Moreover, our analysis identified nuclear factor erythroid 2-related factor 2 (NRF2), a downstream factor of SIRT3, as a significant target in the DHGC network pharmacological analysis. The SIRT3/NRF2/HO-1 signaling pathway acts as an inhibitor of apoptosis, thus we focused our attention on this pathway [20].

To validate the results predicted in network pharmacology, we subsequently induced a mouse AKI model using LPS. LPS is a recognized model drug that triggers cytokine synthesis, secretion, and subsequent inflammatory processes [21]. It is also one of the most important causes of sepsis in the pathogenesis of AKI, leading to a storm of inflammatory factors, increased oxidative stress, inadequate renal perfusion, and ultimately a progressive acute decline in renal function [21]. Creatinine and urea are metabolic end products of creatine, phosphocreatine, and ammonia synthesis [22, 23]. In cases of acute impairment of renal function, creatinine, and urea are not excreted, resulting in increased serum levels (Figure 5C, 5D). The high-dose DHGC group intervention led to decreased serum creatinine and urea levels (Figure 5C, 5D). This may be attributed to Emodin, an active ingredient of Rhubarb that increases glomerular filtration by inhibiting glomerular podocyte apoptosis and endoplasmic reticulum stress [24]. DHGC’s main component, Rhein, possesses various pharmacological activities, such as antibacterial, anti-inflammatory, and anti-ulcer effects, and delays the progression of AKI [2]. Nevertheless, Rhubarb may increase kidney damage due to its toxicity, which should not be ignored [25]. Traditional Chinese Medicine (TCM) theory suggests that drugs with milder medicinal properties are often taken to reduce Rhubarb’s toxicity. Modern pharmacological studies support this TCM theory by demonstrating that the toxicity of Rhubarb is reduced when combined with Licorice [26]. Therefore, DHGC can safely reduce serum creatinine and urea levels in AKI mice by combining Rhubarb and Licorice.

Consistent with previously reported findings, histological analysis of hematoxylin and eosin-stained sections demonstrated that mice treated with lipopolysaccharide (LPS) exhibited renal pathological injury, as evidenced by necrosis, degeneration, and cell swelling of renal tubular epithelial cells [27]. Concomitant apoptosis in the kidney cells of AKI mice was also confirmed by terminal deoxynucleotidyl transferase dUTP nick-end labeling (TUNEL) staining (Figure 6B). However, following gradient intervention with DHGC, the above pathological features were alleviated. The protective effect of DHGC on the kidney was confirmed, while its inhibitory effect on kidney cell apoptosis was established. Apoptosis is induced by various factors, with inflammation being among the most significant factors in AKI [28]. AKI is characterized by acute inflammation, apoptosis, and renal impairment [29]. In AKI patients, elevated levels of nitrogenous substances and serum LPS concentration may contribute to systemic inflammation by increasing the levels of proinflammatory cytokines, such as IL-6, IL-1β, and TNF-α [30]. Prior investigations have demonstrated that Rhubarb decoction exerts an inhibitory effect on inflammation both *in vitro* and *in vivo* [31, 32]. Likewise, we observed that DHGC reduced serum levels of IL-6, IL-1β, and TNF-α (Figure 7), further supporting DHGC’s potential to attenuate apoptosis in AKI mice by suppressing inflammation.

To elucidate the mechanism by which DHGC inhibits apoptosis, we investigated the SIRT3/NRF2/HO-1 signaling pathway based on network pharmacology analysis. Recent evidence has demonstrated that curbing excessive production of reactive oxygen species (ROS) in mitochondria can attenuate renal injury, decrease cytokine release, and suppress renal inflammation [33]. Moreover, mitochondrial autophagy controls mitochondrial mass and mitigates mitochondrial ROS by degrading damaged mitochondria and inhibiting renal apoptosis [34] As reported, the Nrf2/HO-1 signaling pathway has been proven to function as one of the key molecular mechanisms participating in oxidative stress, inflammatory activity, and damaged mitochondria in AKI mice [35–37]. SIRT3 inhibits AKI progression through anti-inflammation, anti-oxidation, anti-apoptosis, and maintenance of mitochondrial homeostasis [38]. Therefore, targeting the reduction of mitochondrial damage (i.e., inhibiting apoptosis) may represent a promising therapeutic strategy against AKI. SIRT3, a member of the Sirtuin family of mammalian histone deacetylases, is predominantly expressed in cellular mitochondria and plays a critical regulatory role in human physiology and various diseases [39]. SIRT3 has been shown to mitigate mitochondrial oxidative damage and apoptosis [19]. Based on network pharmacological analysis, we hypothesized that activation of the SIRT3/NRF2/HO-1 signaling pathway may impede apoptosis in AKI mice. NRF2 mediates genes that encode antioxidative enzymes, antiapoptotic proteins, and detoxifying factors [40]. Additionally, Nrf2 exerts a protective effect against oxidative stress by regulating antioxidative genes, including NQO1 and heme oxygenase-1 (HO-1), which act as antiapoptotic agents [41]. HO-1, which is transcriptionally regulated by Nrf2, serves as an oxidative stress marker that contributes to antioxidant defense and apoptosis prevention [42]. Our data revealed that the SIRT3/NRF2/HO-1 signaling pathway was significantly inhibited in AKI mice due to increased apoptosis in kidney cells (Figure 8). Notably, DHGC exhibited a dose-dependent activation of this signaling pathway, which may represent a pivotal mechanism in inhibiting renal apoptosis and delaying AKI progression.

In summary, this study demonstrated that network pharmacological analysis confirms the broad anti-AKI mechanism of DHGC, which is potentially related to its anti-inflammatory and anti-apoptotic effects on renal cells. Experimental validation confirmed that DHGC alleviates the progression of AKI in mice, as indicated by the reduction of serum inflammatory factors and renal cell apoptosis. DHGC’s therapeutic effect in attenuating apoptosis may be attributed to the activation of the SIRT3/NRF2/HO-1 signaling pathway. These findings provide insight into the potential clinical application of DHGC in the treatment of nephropathy.
